# Stoichiometries of U2AF35, U2AF65 and U2 snRNP reveal new early spliceosome assembly pathways

**DOI:** 10.1093/nar/gkw860

**Published:** 2016-09-28

**Authors:** Li Chen, Robert Weinmeister, Jana Kralovicova, Lucy P. Eperon, Igor Vorechovsky, Andrew J. Hudson, Ian C. Eperon

**Affiliations:** 1 University of Leicester, Leicester Institute for Structural and Chemical Biology and Department of Molecular and Cell Biology, Leicester LE1 9HN, UK; 2 University of Southampton, Faculty of Medicine, Southampton SO16 6YD, UK; 3 University of Leicester, Leicester Institute for Structural and Chemical Biology and Department of Chemistry, Leicester LE1 7RH, UK

## Abstract

The selection of 3΄ splice sites (3΄ss) is an essential early step in mammalian RNA splicing reactions, but the processes involved are unknown. We have used single molecule methods to test whether the major components implicated in selection, the proteins U2AF35 and U2AF65 and the U2 snRNP, are able to recognize alternative candidate sites or are restricted to one pre-specified site. In the presence of adenosine triphosphate (ATP), all three components bind in a 1:1 stoichiometry with a 3΄ss. Pre-mRNA molecules with two alternative 3΄ss can be bound concurrently by two molecules of U2AF or two U2 snRNPs, so none of the components are restricted. However, concurrent occupancy inhibits splicing. Stoichiometric binding requires conditions consistent with coalescence of the 5΄ and 3΄ sites in a complex (I, initial), but if this cannot form the components show unrestricted and stochastic association. In the absence of ATP, when complex E forms, U2 snRNP association is unrestricted. However, if protein dephosphorylation is prevented, an I-like complex forms with stoichiometric association of U2 snRNPs and the U2 snRNA is base-paired to the pre-mRNA. Complex I differs from complex A in that the formation of complex A is associated with the loss of U2AF65 and 35.

## INTRODUCTION

The processes of recognition and selection of 3΄ splice sites (3΄ss) are complex and poorly understood. 3΄ss comprise several distinguishable elements that are recognized directly by RNA-binding proteins: the branch site, a polypyrimidine-rich tract and an AG preceding the 3΄ss itself. The polypyrimidine tract is recognized initially by the protein U2AF65 (1–3), and the YAG/R 3΄ss consensus by U2AF35 (4–7). U2AF65 associates with U2AF35 as a stable heterodimer (1), and interacts with SF1/mBBP (8,9), which recognizes the branch site but is not an essential splicing factor (10–12). The U2 snRNP is recruited later by interactions with SF1 and U2AF65, and base-pairs with the pre-mRNA around the branch site, displacing SF1 (12). It is not clear whether any or all of these sequence-specific interactions with the pre-mRNA occur before or after the transition from recognition to selection of the 3΄ss. A defining characteristic of events involving the recognition of candidate sites is that, if there should be multiple candidate sites per intron, then proteins involved in the recognition of sites would be able to interact with them all and, if the sites were strong, multiple sites on one pre-mRNA molecule might be occupied concurrently. In contrast, only one molecule of factors recruited after selection would associate per intron, regardless of the strength of alternative sites.

The current model for an early stage in which the 3΄ss sequence elements have been recognized is complex E (Early), which accumulates on pre-mRNA incubated in nuclear extracts after depletion of adenosine triphosphate (ATP) (13–16). This complex is committed to splicing (13,14). It contains U2 snRNPs (17–19), but the U2 snRNA does not form psoralen-induced cross-links to the pre-mRNA and is thus thought not to form base-pairs at this stage with the branch site (19). Even though complex E is committed to splicing, it is not clear whether the sites have been selected. In favor of this is evidence from hydroxyl radical probes that the 5΄ splice sites (5΄ss), polypyrimidine tract and branch site are in close proximity with each other and with U2AF65 and U1 and U2 snRNAs (20–22), as if it is a structured complex that involves selected sites. On the other hand, functional assays have suggested that in at least one system the 3΄ss is not irrevocably chosen until complex A (23). Complex A is the first identifiable complex that forms in the presence of ATP; it requires ATP hydrolysis and contains U2 snRNA base-paired to the branch site (24,25). If complex E is a model for the early events of splicing that precede a requirement for ATP, it would be followed in ATP by the formation of pre-spliceosomal complex A.

**Figure 1.**
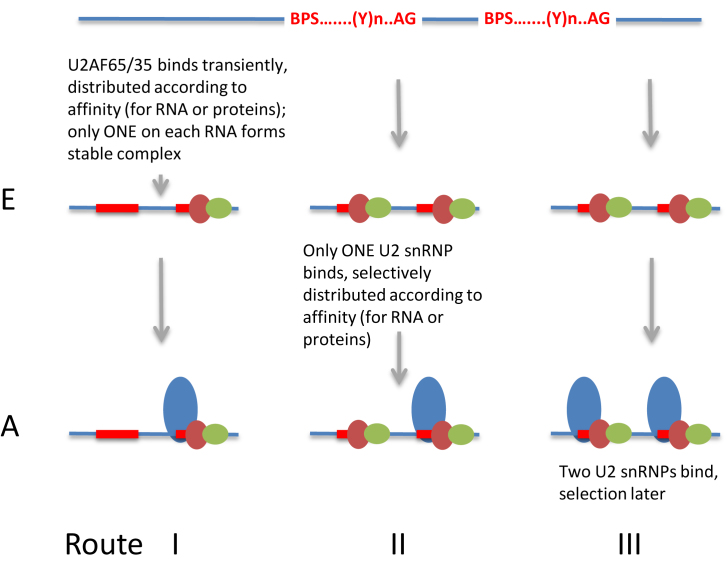
Routes for the processes of recognition and selection of 3΄ splice sites (3΄ss), using as an example a portion of pre-mRNA with two candidate 3΄ss. Blue line, pre-mRNA; red text and boxes, the branchpoint sequence, polypyrimidine tract and 3΄ss; brown oval, U2AF65; green oval, U2AF35; blue oval, U2 snRNP. Route I, U2AF binds according to its affinity to potential sites, but binding is so weak that the average number of molecules bound is <1. Interactions with the 5΄ss result in the formation of complex E, with one stabilized U2AF bound. This is then recognized by a single U2 snRNP when complex A assembles. Route II, U2AF binds strongly to potential sites, so that the 3΄ss is not selected in complex E; U2 binding is initially very weak so that there is on average only one U2 snRNP per intron, and this is incorporated into complex A. Route III, both U2AF and U2 snRNP bind strongly to potential sites and the 3΄ss is not selected by affinity in complex A.

We have outlined three possible routes based on complexes E and A by which recognition could lead to selection (Figure 1). In these schemes, we suggest that U2AF65 and U2AF35 are primary binding factors, like the U1 snRNP at the 5΄ss, that are able to interact directly with all possible sequences. U2AF65 is a strong candidate for such a role as it has two RNA Recognition Motif (RRM)-type RNA-binding domains that confer an independent binding capability with a preference for polypyrimidine tracts (1,26–28), as well as protein or RNA interaction domains (9,29–34). It binds pyrimidine tracts extensively across transcribed regions, with enrichment at 3΄ss (2,3). U2AF35 is generally considered to function as a heterodimer with U2AF65, although U2AF65 is likely to be present in a several-fold excess and U2AF35 homodimers have been observed also (1,3,35–38).

In the first possible route (I, Figure 1), recognition and selection take place when U2AF65/35 binds. The heterodimer may be able to sample all candidate sequences, binding transiently such that no more than one candidate site is occupied at any moment. If an occupied site were to encounter a 5΄ss-bound U1 snRNP or SF1/mBBP at the branch site, then a stable complex E might form that would provide a unique recruitment site for U2 snRNPs. The level of U2AF65 binding is known to be a determinant of U2 snRNP binding and thus splicing (26,39,40). Fittingly, a number of proteins that inhibit splicing reduce U2AF65 binding, usually by direct competition (2,41–44). The use of potential sites would be distributed according to their occupied lifetimes. Particular sites might be favored if, for example, SR proteins bound to exonic splicing enhancer sequences in the 3΄exon interacted with U2AF35 and stabilized the heterodimer (45). However, the connection between enhancer activity and U2AF65 recruitment is not unambiguous (46–55).

There are arguments against route I. In particular, one study has shown that 3΄ss selection is not fixed in complex E (23). Not enough is known to explain the discrepancy with the observation of commitment to splicing in complex E. Moreover, in yeast, single molecule FRET data suggest that the 5΄ss and 3΄ss sites are not within 5–10 nm in yeast early complexes (56). Finally, there is no evidence of any mechanisms by which interactions of U2AF65/35 with SR proteins or U1 snRNPs (19,52,57–60) might be prevented from stabilizing more than one U2AF heterodimer. Given that there are many potential 3΄ss sequences in long introns (3,61), it is quite plausible that multiple stable U2AF65/35 complexes might form. In such a case, selection might take place at the level of U2 recruitment (route II). This might happen if the U2 snRNP's interaction with U2AF65/35 were weak and required stabilization by interactions with a single 5΄ss-U1 snRNP complex. This would fit the evidence that the 3΄ss is fixed in complex A (23). Alternatively, multiple U2 snRNPs might bind and selection take place later (route III). There is no evidence yet to indicate whether two or more U2 snRNPs are permitted to associate with a single intron.

The clearest method for discriminating among these potential routes for 3΄ss selection is to determine the number of molecules of U2AF and U2 snRNP bound in complexes E and A to a molecule of pre-mRNA that contains alternative strong 3΄ss. However, standard ensemble methods for analyzing protein–RNA interactions are unable to give more than, at best, a mean value. The mean is in itself of little use, because it might hide a very wide range or multiple discrete populations. For these reasons, we have used single molecule fluorescence methods (62–64). We conclude that there is no single route for selection and we propose an alternative state, complex I, as a model for the first steps of spliceosome assembly.

## MATERIALS AND METHODS

### Sample preparation

Pre-mRNA for single molecule experiments was transcribed *in vitro* as described (62) with the exception that guanosine 5΄-O-monophosphorothioate was included for initiation of transcription to enable subsequent labeling with Cy5-maleimide (Lumiprobe) (65). Nuclear extracts were prepared from HEK293T cells transfected with plasmids expressing either mEGFP-U2B″, mEGFP-SF3A3 or both mEGFP-U2AF65 and mCherry-U2AF35; these were pre-incubated for 15 min in the presence or absence of ATP and phosphocreatine, pre-mRNA was added to 62.5 nM and reactions were incubated, as described (64). The reactions were diluted and immediately applied to cover slips. When present, a cocktail inhibiting protein phosphatases (PhosSTOP, Roche) was added at a 2× concentration prior to the pre-incubation for ATP depletion. The 2΄-O-methyl oligonucleotide complementary to U6 snRNA was used as described (64).

### Data acquisition and analysis

The cover slips were 22 × 50 mm, #1 (Menzel-Gläser), washed, sonicated, dried under vacuum and cleaned five times in an argon plasma for 5 minutes each time (Weinmeister *et al.*, submitted). Fluorescence was detected using a home-built objective TIRF microscope, and data analyzed from the central 250 × 250 pixels of the 512 × 512 pixel charge-coupled device (CCD) chip. The position of the reflected laser beam, determined by a quadrant photodiode, was used as active feedback to control the microscope focus. Data was initially collected from Cy5-labeled RNA excited at 640 nm until the fluorophores bleached, and then from mCherry (if used), excited at 561 nm, again until the fluorophore had bleached and finally from mEGFP excited at 488 nm until that bleached too. The experiments with U2AF35 and U2AF65 involved collection of data from both proteins in the same reaction mixture. Spots were detected and accepted based on the Gaussian distribution of their intensities. Chromatic aberrations that affect colocalization were corrected by a linear transformation. Step detection in the bleaching profile was based on a Bayesian algorithm (66). A complete description is given in Weinmeister *et al.* (submitted).

Each frequency histogram shows the result of measurements made on 200–800 individual RNA molecules (average 425), collated from around 50 fields acquired from each sample. The error bars are the square root of the variance of the binomial probability that an RNA spot will be associated with the given number of protein bleaching steps. Most of the experiments were done once, and the general conclusions of most experiments were borne out by repeats with different transcripts. Where experiments were repeated three times to accumulate data, the standard deviation for the colocalization was no more than 13% of the mean value and was generally less than 10% (data not shown).

The expected distributions for a single site were taken directly from the observed distributions of labeled protein spots bleaching in one step or two steps in splicing conditions but in the absence of pre-mRNA. In the case of U2AF35 and U2AF65, these measurements were made in the presence and absence of ribonuclease. Measurements were made in the presence or absence of ATP. The expected distributions of steps if two sites were fully occupied were calculated using the data above and the proportions of functional protein labeled, measured by affinity purification on biotinylated pre-mRNA. Chi-square tests were done on classes where the expected number was ≥5. The proportions of pre-mRNA occupied at no sites, one site and two sites were estimated by varying the proportions and the total number of spots considered accessible in principle, with minimization of the chi-square difference from the actual results.

### Assay of U2AF35 activity

HEK293T cells were depleted of U2AF35 using two hits of small interfering RNA U2AF35ab, as described (67). Rescue and reporter plasmids were added 24 h after the second hit at a concentration of 250 and 150 ng/ml, respectively. The transfected cells were harvested 48 h later. Total RNA was extracted as described and reverse transcribed with MMLV (Promega) and oligo-dT_15_. RNA products were visualized as described (67). Immunoblotting was carried out with antibodies against U2AF35 (Protein Tech Group, 10334-1-AP) and GFP (Abcam, ab290).

### Splicing and analysis of complexes

Extracts were prepared, splicing assayed and complexes analysed by gel electrophoresis as described previously (64). RNase H protection and psoralen crosslinking assays were done as described (68,69) using globin-based substrates as previously described (70). Affinity purification to measure the relative levels of fused and endogenous protein bound to RNA was done with NeutrAvidin-agarose beads (ThermoFisher) from 40 μl splicing reactions containing biotinylated RNA at 50 nM. Deoxyoligonucleotides E15 and L15 (71) were added in control reactions with the mEGFP-U2B″ to stimulate RNase H digestion of U2 snRNA. Proteins were detected by western blotting, using primary antibodies against U2AF35 (Proteintech 60289), U2AF65 (MC3) (72), U2B″ (Proteintech 13512), GFP (Proteintech 66002) and dsRed/mCherry (Clontech 632496). Fluorescent secondary antibodies were used against mouse IgG (IRDye 680LT, LI-COR 926–68020) and rabbit IgG (IRDye 800CW, LI-COR 926–32211). Concentration standards were recombinant proteins, mCherry (Origene TP790040) and GFP.

## RESULTS

To enable the binding of individual molecules of U2AF65 and U2AF35 to pre-mRNA in nuclear extracts to be detected, U2AF65 and U2AF35 were co-expressed as N-terminal fusions with mEGFP and mCherry respectively. U2 snRNP was labeled by expressing either U2B″ or SF3A3, components of the U2 snRNP and its associated SF3a complex respectively, as fusions with mEGFP. Fluorescent protein fusions with U2AF65, U2AF35 and U1A (a close relative of U2B″) in this vector have been shown previously to be properly localized in the cell and interact as expected with other proteins or to be functional (35,64,73). We confirmed that the mCherry-labeled U2AF35 was able to rescue correct splicing of intron 1 of the insulin gene, which is mis-spliced after depletion of U2AF35 (67) (Supplementary Figure S1). Active nuclear extracts were prepared from HEK293T cells expressing either mEGFP-U2B″, mEGFP-SF3A3 or both mEGFP-U2AF65 and mCherry-U2AF35 and the absolute and relative levels of functional labeled protein and their extent of dimerization were measured using western blotting after affinity purification on RNA and single molecule methods, respectively (Table 1 and Supplementary Figure S2). The total concentrations of U2AF65 and U2AF35 in the nuclear extract are approximately 1.88 and 0.27 μM, respectively. A relative excess of U2AF65 over U2AF35 was apparent also in a recent estimate of their concentrations in HeLa cells (1.2 and 0.5 μM respectively) (37).

**Table 1.** Concentrations and properties of expressed fluorescent proteins

	mCherry-U2AF35	mEGFP-U2AF65	mEGFP-U2B″
Concentration in splicing (μM)	0.2	1.3	1.4
% labeled:unlabeled on RNA aff. purifn.	75:25	69:31	49:51
Molecules bleaching in 2 versus 1 steps, −ATP	33:189*	33:243*	39:162
Molecules bleaching in 2 versus 1 steps, +ATP	21:218*	25:186*	30:252

Concentrations were calculated from western blotting of nuclear extract alongside recombinant protein; the percentages labeled or unlabeled (endogenous) recovered after affinity purification on biotinylated RNA were also calculated by western blotting; the numbers of molecules recorded as bleaching in one or two steps in the absence of exogenous RNA are shown. *, done in the presence of ribonuclease.

In the first experiments, measurements were made to establish the stoichiometries with which these components bound to a pre-mRNA containing no alternative sites in conditions forming complexes E and A. A β-globin-derived transcript with one intron and a consensus 5΄ss (GloC) (64) was labeled at the 5΄ end with Cy5-maleimide, a strategy used previously to study the dynamics of spliceosome assembly (65). The RNA was incubated in the nuclear extracts and the mixtures were then diluted and immediately applied to a glass cover slip. Time courses of fluorescence of proteins colocalized in a spot with RNA were analyzed to detect bleaching of the individual fluorescent protein molecules associated with an RNA molecule. The number of bleaching steps in one spot reveals the number of fluorescent protein molecules bound to an individual molecule of pre-mRNA.

### U2AF35 binds singly to pre-mRNA in complex E but U2 snRNP shows no discrete stoichiometry

**Figure 2.**
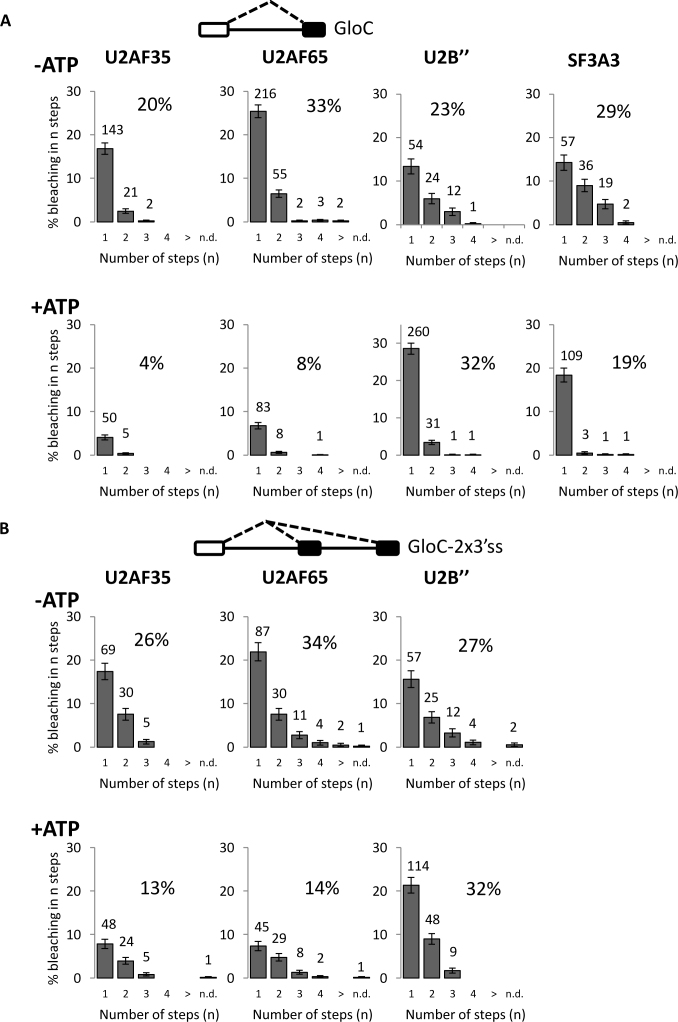
ATP-dependent changes in the association of U2AF35, U2AF65 and U2 snRNP with pre-mRNA containing one or two 3΄ss. (**A**) Frequencies (%) of globin-derived pre-mRNA (GloC) molecules showing bleaching of colocalized labeled protein in **n** steps after incubation in nuclear extracts containing either mCherry-U2AF35 and mEGFP-U2AF65, mEGFP-U2B″ or mEGFP-SF3A3. Each bleaching step corresponds to bleaching of one molecule of fluorescent protein associated with one molecule of pre-mRNA. Thus, the number of bleaching steps detected indicates the number of molecules of the specified fluorescent protein bound to a molecule of RNA. The number above each bar indicates the number of complexes in which complete bleaching was achieved in 1, 2, 3, etc., steps, and hence the number of complexes in which there were 1, 2, 3, etc., molecules of fluorescent fusion protein. > refers to complexes where more than four bleaching steps were measured; n.d. represents complexes where the number could not be determined. The percentage value above each histogram is the percentage of labeled pre-mRNA molecules that were associated with any fluorescent protein. −ATP, nuclear extracts depleted of ATP; +ATP, extracts containing ATP and an oligonucleotide complementary to U6 snRNA that blocks progression beyond complex A. The pre-mRNA is represented above the histograms. The white box represents an exon with a 5΄ss, the black box an exon with a 3΄ss and the line in between is the intron. Potential splicing pathways are shown as dashed lines. (**B**) Frequencies of complexes showing bleaching of proteins colocalized with pre-mRNA containing two 3΄ss (GloC-2 × 3΄ss) in nuclear extracts either −ATP or +ATP, as above.

Depletion of ATP by pre-incubation of the extract at 30°C enables only complex E to assemble (74). Under these conditions, 20% of the surface-bound molecules of pre-mRNA colocalized with mCherry-U2AF35, and in most of these complexes the mCherry bleached in a single step, indicating that only one labeled protein was associated (Figure 2A). In 21 of the 164 complexes bleaching took place in two steps. This is about the same proportion as found for free mCherry-U2AF35 in the absence of RNA, due to dimerization (Table 1). Assuming that the same proportion of molecules is present as a dimer on the RNA, we conclude that a single site in the pre-mRNA was bound by mCherry-U2AF35 (single occupancy; χ^2^, *P* = 0.45). For U2AF65,55 of 278 complexes bleached in two steps (Figure 2A). Taking into account the proportion of U2AF65 bound to RNA that is labeled (Supplementary Figure S2) and the level of RNA-independent dimerization (Table 1), this does not fit single occupancy (χ^2^, *P* = 3 × 10^−5^). Instead, the best fit to our model suggests that around 12% of complexes were bound at two sites by U2AF65. Finally, in the extract expressing mEGFP-U2B″, a number of complexes showed bleaching in two or even three steps. A very similar result was found when the U2-associated protein SF3A3 was labeled instead. Again, taking into account dimerization, we estimate that over half of the complexes contained two or more U2 snRNPs. We conclude that the only component to show only single occupancy was U2AF35, and that U2 snRNP in particular associated less stringently.

To test whether the slight propensity of U2AF65 toward double occupancy on some molecules of pre-mRNA was the result of binding to the RNA rather than protein interactions, nuclear extracts were prepared from cells co-transfected with mCherry-U2AF65 and mEGFP-labeled U2AF65 variants lacking the N-terminal RS domain or the C-terminal UHM domain, or both (Supplementary Figure S3A). Despite similar levels in the nuclear extracts, only the variant that contained the two RNA-binding domains bound to the pre-mRNA in the absence of ATP, and its association pattern was very similar to that seen with the wild-type protein (Supplementary Figure S3B). This suggests that the normal binding pattern requires only the RNA-binding activity of U2AF65 and is not contingent upon protein interactions, supporting a role for U2AF65 in the primary recognition of candidate sites.

### All three components bind with a fixed stoichiometry in ATP, but U2AF binding is reduced

Figure 2A also shows results in extracts containing ATP and an oligonucleotide that anneals to U6 snRNA and prevents progression beyond complex A (22) (Supplementary Figure S4A and B). All three components (U2AF35, U2AF65 and U2 snRNP, whether labeled via U2B″ or SF3A3) showed single occupancy of the pre-mRNA (χ^2^, *p_U2AF35_* = 0.94, *p_U2AF65_* = 0.37, *p_U2B_*_″_ = 0.98). In the case of U2AF35, the distribution was the same as in the absence of ATP (χ^2^, *p_U2AF35_* = 0.41, *p_U2AF65_* ≤ 0.01, *p_U2B_*_″_ ≤ 0.01).

The results also showed very striking changes in the proportion of RNA molecules colocalized with the fluorescent proteins. The level of association with U2AF35 and U2AF65 was sharply reduced in the presence of ATP, whereas the levels of U2 snRNP and SF3a binding were less affected. This suggests that the consolidation of U2 snRNP in complex A was associated with displacement of U2AF35 and U2AF65. However, co-expression of mCherry-U2AF65 and mEGFP-U2B″ and analysis under conditions supporting complex A formation showed that both components could bind concurrently and there was no evidence of mutual exclusion (Supplementary Figure S5). It is possible that there are two populations in the presence of ATP, representing an early stage with U2AF bound and a later stage where it is being displaced. We conclude from Figure 2A that there is stoichiometric binding of U2AF35 in complex E and of all components in complex A. This means that it should be possible to distinguish between recognition and selection by determining whether the presence of alternative sites affects the numbers of components bound.

### Two molecules of U2AF35 and U2 snRNPs can bind pre-mRNA with strong alternative 3΄ss

We measured binding to a variant of the globin substrate in which the strong 3΄ss had been duplicated (GloC-2 × 3΄ss; Figure 2B). In the absence of ATP, there was significantly increased binding of a second molecule of U2AF35 compared with the pre-mRNA containing a single 3΄ss in Figure 2A (χ^2^, *p*_(_*_2_*_sites_ _= 1 site)_ << 0.01), but there was only a smaller change in U2AF65 stoichiometry and no change in the frequency distribution for U2 snRNP association (χ^2^, *p_2_*_sites = 1 site_ = 0.94). If complexes formed in the absence of ATP are valid models for the first stage of pre-mRNA recognition, then the results are consistent with routes II or III (Figure 1) for U2AF35, but U2 snRNP association is unrelated to the number of potential sites.

In the presence of ATP there was a very significant shift for all components from occupation of one site on GloC toward occupation of two sites. We estimate that roughly equal numbers of RNA molecules show single and double occupancy with all three components, consistent with the evidence from native gel electrophoresis (Supplementary Figure S6A). We infer that, in the presence of ATP, even U2 snRNP can associate with potential rather than selected sites, as in route III (Figure 1).

This substrate spliced poorly (Supplementary Figure S6B). To test whether this is the result of double occupancy, a competitor RNA oligonucleotide containing the sequence of the polypyrimidine tract and 3΄ss was added to reduce the availability of unbound factors. If the hypothesis were correct, there would be reduced double occupancy and an otherwise paradoxical increase in splicing efficiency; if incorrect, the competitor would be expected to decrease the efficiency of splicing. The results show that levels of splicing were increased several-fold (Supplementary Figure S6C). Moreover, single molecule analyses in the absence of ATP confirmed that the oligonucleotide reduced the proportion of pre-mRNA molecules with double occupancy by U2AF35 (Supplementary Figure S6D), whereas it had no effect on the binding of U2AF65 or U2 snRNP (data not shown).

We conclude that two strong potential alternative sites can be occupied concurrently in the presence of ATP on some molecules of pre-mRNA, as in Figure 1, route III. This is reminiscent of the binding of U1 snRNPs (64), and strongly suggests that all three components could be involved in the initial recognition of candidate sites. The relatively low level of splicing compared with the parental construct with a single 3΄ss and the competition experiment in which reduced occupancy by U2AF35 increased splicing suggest that routes II and III might be disadvantageous or even non-productive.

### Sequential formation of complex A on two introns

**Figure 3.**
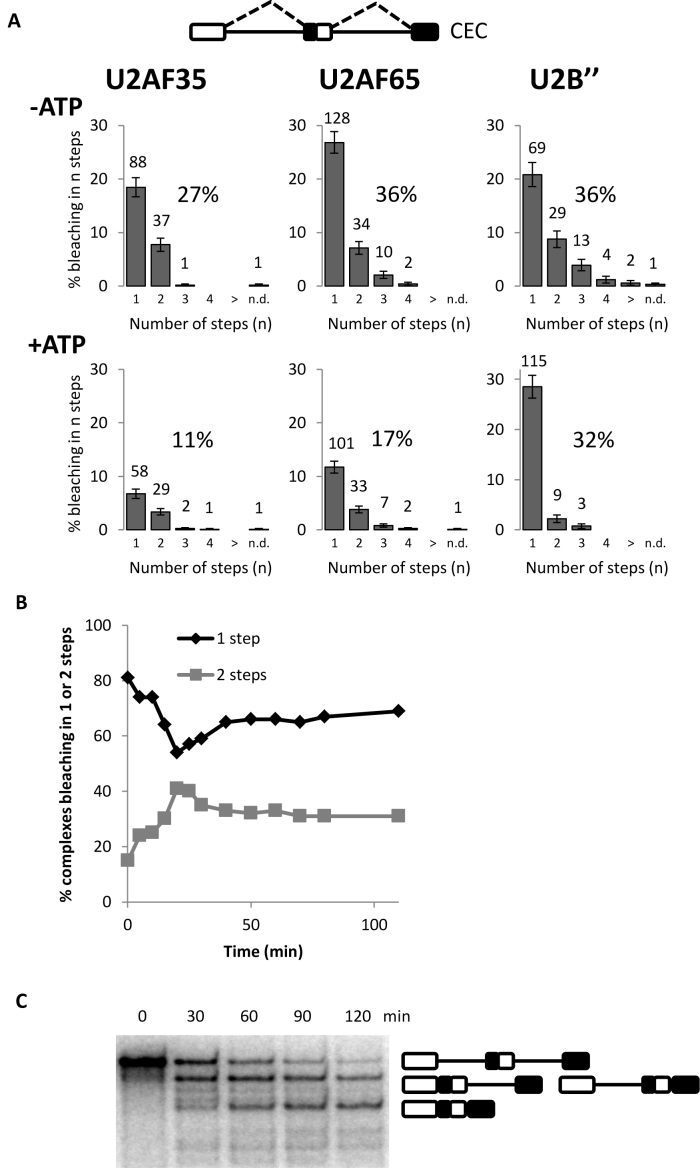
Association of single U2 snRNPs on globin-derived pre-mRNA with two introns (CEC) under conditions favoring accumulation of complex A. (**A**) Frequencies of pre-mRNA complexes in which fluorescent factors bleached in the indicated number of steps after incubation of the RNA in nuclear extract either depleted of ATP (−ATP) or in the presence of ATP and oligonucleotide complementary to U6 snRNA (+ATP). (**B**) Frequencies of bleaching of mEGFP-U2B″ in one or two steps for CEC complexes formed after incubation for the times indicated in the presence of ATP without oligonucleotide complementary to U6 snRNA. (**C**) Time course of splicing of CEC pre-mRNA. The mRNA products are indicated.

It was not clear from the experiment in Figure 2B whether the presence of molecules bound by only one molecule of U2AF or U2 snRNP was the outcome of stochastic events or whether there were any barriers to double occupancy. Thus a pre-mRNA was tested that contained two identical introns and would be expected to exhibit no barriers to occupancy by two molecules of any splicing factor (Figure 3A, CEC; (64)). The distributions of binding in ATP-depleted extracts were insignificantly different from those seen with duplicated 3΄ss (Figure 3A, c.f. 2B). However, when ATP was present but the progression of complexes beyond complex A was blocked by an oligonucleotide as usual, only one U2 snRNP bound (Figure 3A). In the absence of a blocking oligonucleotide, the distribution fitted binding by two U2 snRNPs (data not shown; χ ^2^, *p_U2B_*_″_ = 0.4). To investigate this further, samples were taken from an uninhibited splicing reaction at various time intervals and U2 snRNP binding analyzed. Initially, bleaching took place predominantly in a single step, but with increasing time the proportion of molecules in which bleaching took place in two steps rose, and then it fell slightly (Figure 3B). A time course of a normal splicing reaction showed that the splicing of one intron preceded that of the other (Figure 3C). We conclude that formation of pre-spliceosomal complex A on one intron initially inhibited the association of U2 snRNP, but not U2AF35, with the other intron.

### The effects of ATP on U2 snRNP binding can be recapitulated by inhibition of phosphatases

**Figure 4.**
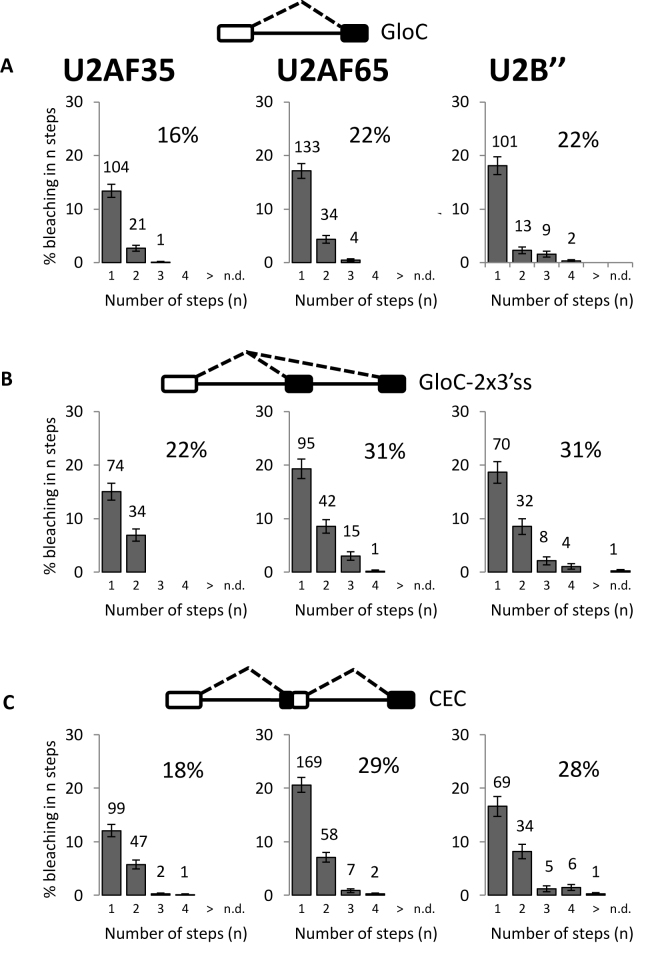
U2 snRNP association in the absence of ATP is the same as in the presence of ATP when phosphatases are inhibited. Histograms show the numbers of steps in which U2AF35, U2AF65 and U2 snRNP associated with pre-mRNA bleach after incubation in nuclear extracts depleted of ATP but containing phosphatase inhibitors. (**A**) GloC pre-mRNA. (**B**) GloC-2×3΄ss pre-mRNA. (**C**) CEC pre-mRNA.

The results in Figures 2 and 3 showed a major difference in the association of U2 snRNPs between conditions in which ATP had been depleted (complex E) or added (complex A). Significantly, surplus U2 snRNP was absent in the presence of ATP. ATP might contribute in at least two ways: by mediating structural effects, such as remodeling complexes or enabling helicase activity, or by maintaining protein kinase activity to counteract phosphatases. To distinguish between these two possibilities, a cocktail of inhibitors of protein phosphatases 1 and 2A was included during ATP depletion (Figure 4). These affect splicing after spliceosome assembly (75), but do not affect complex E formation (Supplementary Figure S7A). The inhibitors did not greatly affect the association of U2AF35 and U2AF65 (Figures 2A and 4A, χ^2^, *p_U2AF35_*, _E+Inh versus E_ = 0.18; *p_U2AF65_*, _E+Inh versus E_ = 0.98; Figures 2B and 4B, χ^2^, *p_U2AF35_*, _E+Inh versus E_ = 0.06; *p_U2AF65_*, _E+Inh versus E_ = 0.35; Figures 3A and 4C, χ^2^, *p_U2AF35_*, _E+Inh versus E_ = 0.49; *p_U2AF65_*, _E+Inh versus E_ = 0.04;). However, there was a striking shift in U2 snRNP binding to the pattern seen in the presence of ATP, with a single molecule per transcript (χ ^2^, *p_U2B″,_*_E+Inh versus A_ = 0.8). The same result was seen using SF3A3 as an alternative indicator for U2 snRNPs (data not shown). Corresponding results were seen with the pre-mRNA containing two 3΄ss, where the distribution of U2 snRNP matched that seen in the presence of ATP (Figures 2B and 4B) (χ ^2^, *P* = 0.6) and was consistent with occupation of two sites (χ^2^, *P* = 0.2). With two introns (CEC, Figure 4C), where in complex A only one U2 snRNP bound, protein phosphatase inhibition produced a pattern consistent with binding by two U2 snRNPs (χ^2^, *P* = 0.2). Moreover, irradiation after incubation with AMT-psoralen showed that U2 snRNA could be cross-linked to the pre-mRNA if protein phosphatases are inhibited, indicative of base-pairing to the branch site (Supplementary Figure S7B). This has previously been considered to be a hallmark of complex A formation, requiring ATP (see ‘Discussion’ section). We conclude that stoichiometric U2 snRNP recruitment and even branch site base-pairing can occur without ATP hydrolysis if there is protection against protein dephosphorylation.

### Weaker alternative 3΄ss show inhibition of ATP-dependent switch from U2AF to U2 snRNP binding

**Figure 5.**
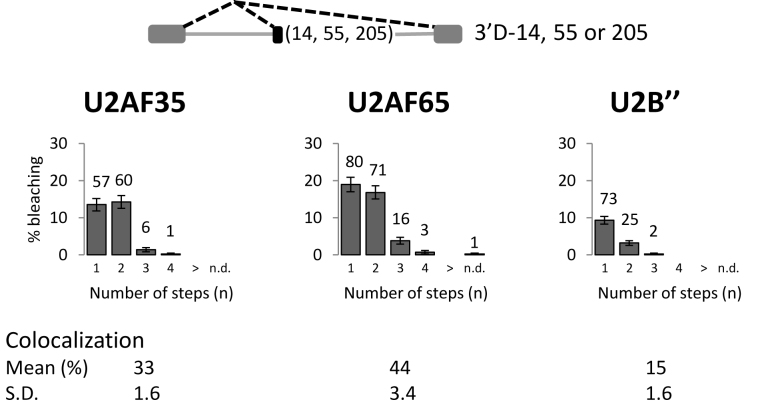
Association of 3΄ss factors with pre-mRNA derived from β-globin intron1 with duplicated 3΄ss, the upstream 3΄ss being adjacent to a portion of the 3΄ exon (black) comprising 14, 55 or 205 nucleotides. (**A**) Frequencies of complexes formed in the presence of ATP and oligonucleotide complementary to U6 snRNA. The results shown are from 3΄D-14, with the means and standard deviations of colocalization from experiments with all three constructs shown below.

The GloC-derived pre-mRNAs used for the experiments in Figures 2–4 contained a consensus 5΄ss and the parental construct splices very efficiently (Supplementary Figure S4). To test whether this is a factor in the formation of complexes consistent with route III by a significant proportion of molecules with alternative 3΄ss in complex A conditions (Figure 2B), we tested alternative model substrates containing duplicated 3΄ss. These pre-mRNAs contained duplications of the human β-globin intron 1 3΄ss, the proximal site being adjacent to 14, 55 or 205 nucleotides of exon 2 (Figure 5) (76). The length of the proximal exon has been reported to affect the choice of 3΄ss, but in our experiments splicing was very inefficient and exclusively to the distal site (Supplementary Figure S7C). Spliceosome assembly was also very weak (Supplementary Figure S7D). In the presence of ATP the patterns of binding of U2AF and U2 snRNPs to the three pre-mRNAs were very different from those seen with GloC-2 × 3΄ss. They showed a higher level of binding and double occupancy by U2AF35 and 65 and a much reduced level of U2 snRNP binding (Figure 5, c.f. 2B), in line with the low levels of complex A formation. However, we estimate that approximately one-third of the U2-containing complexes are associated with two U2 snRNPs. The distributions are shown in Figure 5 only for 3΄D-14, since all three transcripts gave very similar distributions, but the means and standard deviations for the colocalizations are shown for the three transcripts. We conclude that these transcripts are inhibited in progressing from the first ATP-dependent complex dominated by U2AF binding to the second complex in which U2 snRNP is predominant, but that even so some molecules follow route III.

### Sequestration of U1 snRNPs stimulates association but inhibits progression

**Figure 6.**
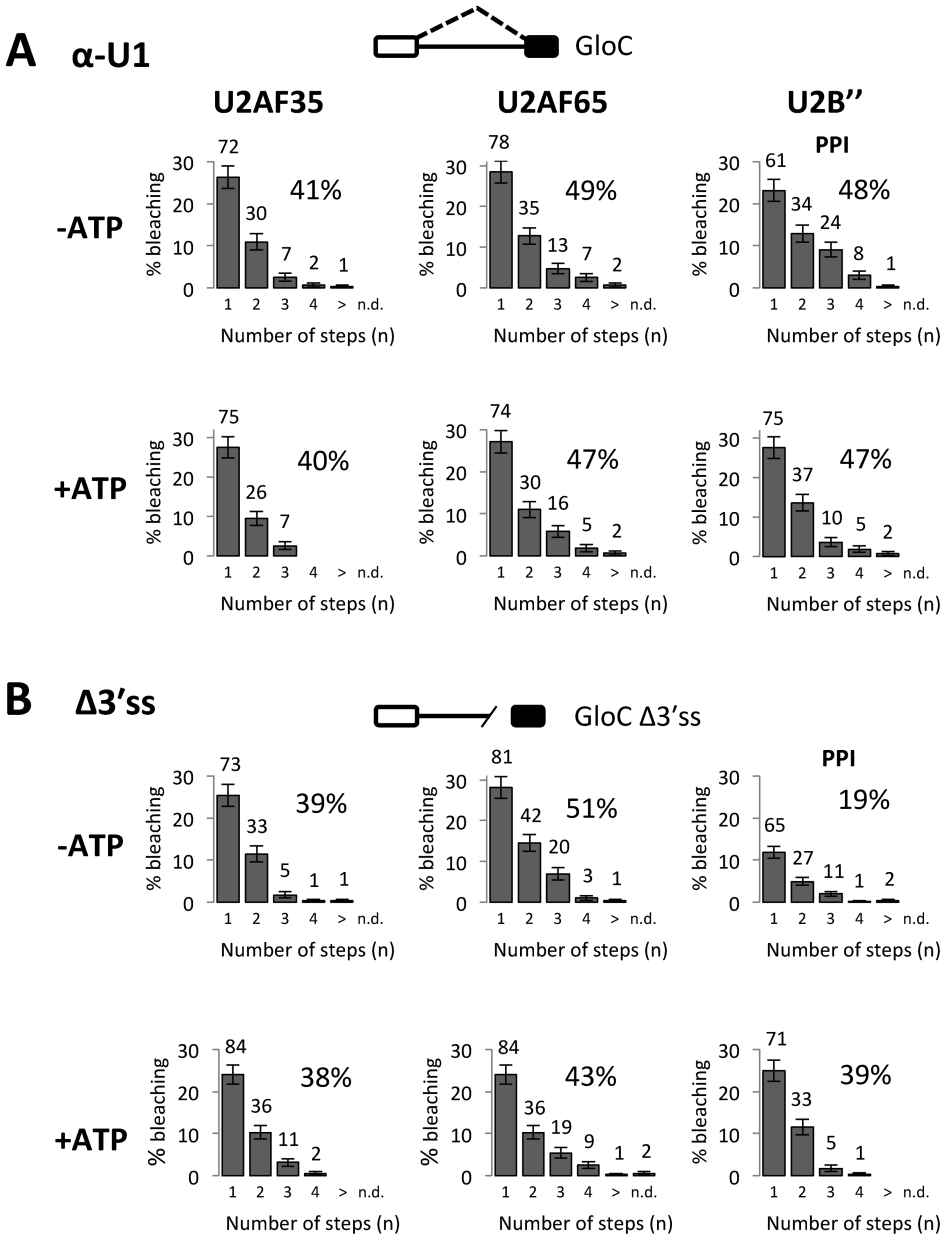
Roles of U1 snRNPs and 3΄ss sequences in limiting the association of factors with pre-mRNA. (**A**) Effects of a 2΄-O-methyl oligonucleotide complementary to the 5΄ end of U1 snRNA on complexes formed in nuclear extract either (top) after depletion of ATP, with protein phosphatase inhibitors (PPI) present in the extract containing mEGFP-U2B″ or (bottom) in the presence of ATP. (**B**) Effects of removing a region of the pre-mRNA including the branch site and the 3΄ss in nuclear extracts after ATP depletion (top) or in the presence of ATP (bottom).

If U2AF and U2 snRNPs are involved in primary recognition, then binding would be expected to be independent of other constitutive components. However, it has been suggested previously that the 3΄ss components are recruited by U1 snRNPs or SR proteins (see Introduction). To test this, U1 snRNPs were sequestered with a 2΄-O-methyl oligonucleotide complementary to the 5΄ end of U1 snRNA (64). Contrary to expectation, U1 inactivation resulted in a 2-fold increase in the level of colocalization of the pre-mRNA with U2AF35, U2AF65 and, even in the presence of protein phosphatase inhibitors, U2 snRNPs (Figure 6A, c.f. Figures 1A and 3A). Moreover, U1 inactivation increased the proportions of complexes bleaching in two, three or four steps. The increased association of U2 snRNP after inhibition of U1 snRNP was surprising, because stable recruitment of U2 snRNP to the branchpoint sequence of such globin substrates requires the 5΄ end of U1 snRNA (70) (Supplementary Figure S8). Incubation in ATP made little difference to the very high levels of association (Figure 6A). Clearly, both protein phosphorylation and U1 snRNPs are required for the formation of stoichiometric complexes.

### Removal of 3΄ss sequences also stimulates association with the pre-mRNA

The most likely reason for the dependence on U1 snRNP is that the association of surplus proteins is prevented by the formation of complexes involving the 5΄ and 3΄ss. The other likely determinants of binding are the conserved 3΄ss signals themselves. The most striking effects were seen when all of the 3΄ss signals were removed from the pre-mRNA. As with the inactivation of U1 snRNP, the levels of U2AF binding in the absence of ATP were increased (Figure 6B), and there was a marked increase in the proportions of complexes bleaching in two or three steps. In the presence of ATP, the deletion prevented the normal loss of U2AF binding and the switch to a single U2 snRNP. We conclude from the effects of U1 snRNP sequestration and mutagenesis of the 3΄ss that the ability to form complexes involving the 5΄ and 3΄ss is required for stoichiometric association.

### Involvement of 3΄ss factors in the regulation of *Tpm1* and SMN2 splicing

**Figure 7.**
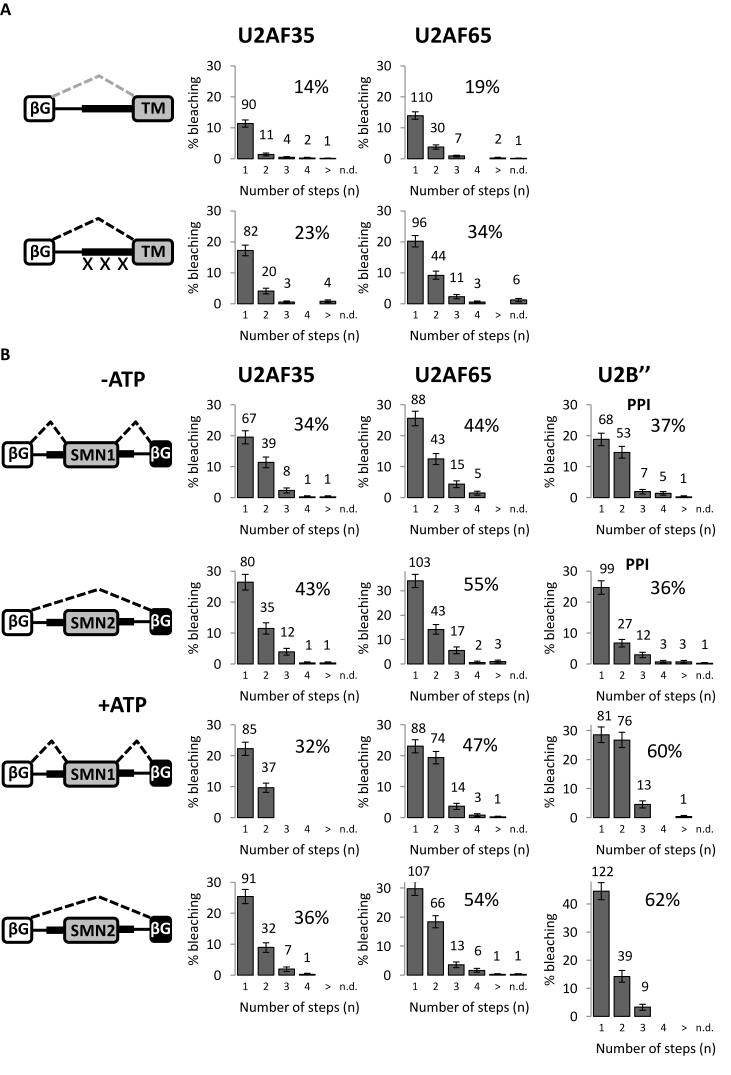
Effects of repressors and activators on recruitment of 3΄ss factors. (**A**) Effects of mutations (crosses) in PTB-binding sites on association of U2AF35 and U2AF65 with pre-mRNA containing the PTB-regulated exon 3 of tropomyosin (*Tpm1*). The construct TM1 contained the 5΄ exon and adjacent 65 nts of the intron of Glo (64), fused to 314 nts of *Tpm1*, comprising 188 nts of intron 2 and the whole of exon 3. In TM4, the three UCUU motifs had been mutated to UUUU (62). The extract had been pre-incubated to remove ATP. (**B**) Association of 3΄ss factors with pre-mRNA containing exon 7 of SMN1 or SMN2. Frequencies of bleaching steps are shown for each pre-mRNA incubated either in nuclear extract pre-incubated to deplete ATP (upper part) or in nuclear extract in conditions favoring the accumulation of complex A.

The results so far present a radical new picture of the early stages of splicing that could have been revealed only by single molecule methods. The super-stoichiometric binding of U2 snRNPs in complex E, the loss of U2AF in the presence of ATP, the increased association and super-stoichiometry after U1 snRNP inhibition or 3΄ss removal and the effect on stoichiometry of inhibiting phosphatases are all unexpected findings, and inevitably raise questions as to whether the method itself introduces artefacts. While ensemble methods are uninformative about stoichiometries, the overall levels of binding of 3΄ss components have been measured in some systems. This provided an opportunity to determine whether the single molecule and ensemble results are consistent. We analyzed two systems in which the binding of U2AF or U2 snRNPs was known to respond to mutations. One is exon 3 of *Tpm1*, which is strongly suppressed by polypyrimidine tract binding protein (PTB) and activated by mutations in probable PTB binding sites in the extended polypyrimidine tract and in the downstream intron (62,77,78). In the absence of the downstream intron, the interpretation is that PTB competes with U2AF binding, and that the mutations reduce the competition. *Tpm1* exon 3 and its upstream intron sequences, with the AA to GG mutation in the branch site required for repression in HeLa cells (79), were used to replace corresponding sequences in the 3΄ half of the standard globin pre-mRNA. Splicing was stimulated about two-fold by the polypyrimidine tract mutations (Supplementary Figure S9). Single molecule analysis showed that the levels of U2AF35 and U2AF65 colocalization were unusually low in the absence of ATP but increased 1.6- and 1.8-fold, respectively, by the activating mutations (Figure 7A). Moreover, a higher proportion of complexes on the activated pre-mRNA contained two molecules of U2AF65, suggesting multiple binding to the long (∼50 nt (80)) upstream polypyrimidine tract.

The other system tested was exon 7 in the genes SMN1 and SMN2, where a single nucleotide difference enhances exon skipping in SMN2. Using pre-mRNA containing three exons and two introns (two exons from globin flanking SMN exon 7), which reproduces the splicing patterns seen *in vivo* (54,81), little difference was seen in U2AF binding between SMN1 and SMN2 in the absence of ATP (Figure 7B), whereas in the presence of phosphatase inhibitors (-ATP) there was a much higher level of binding by two U2 snRNPs on SMN1 RNA. In the presence of ATP, there was a slightly higher level of binding by two molecules of U2AF65 and, again, a much higher level of binding by two U2 snRNPs on SMN1 compared with SMN2. Unlike CEC (Figure 3), assembly on these transcripts with two introns does not appear to be trapped once one U2 snRNP has assembled. We conclude that the major difference between the 3΄ss of exon 7 in SMN1 and SMN2 is in the recruitment of U2 snRNP, in line with previous findings (55).

## DISCUSSION

The purpose of this research was to establish the pathways by which the key components involved in the early steps of splicing at the branch site and the 3΄ss recognize and select candidate sites, and in particular to establish whether they sample all candidate sites or are directed to a pre-selected site by other factors. In addition, it was important to establish whether it is plausible to analyze complexes formed in the absence of ATP (such as complex E) as models for the normal process of assembly, the test principle being that the absence of ATP should only affect ATP-dependent processes and not the states of the components.

Our observations can be summarized thus:

- In the absence of ATP, the numbers of molecules of U2AF35 bound to the pre-mRNA depend strongly on the number of *3*΄ss (Figure 2). Thus, U2AF35 can bind via routes II or III as well as route I (Figure 1). U2AF65 shows some non-stoichiometric association.
- In contrast, U2 snRNP association is super-stoichiometric in the absence of ATP, and independent of the number of candidate sites (Figure 2). This is a result of protein dephosphorylation. Inhibition of phosphatases results in stoichiometric association and ATP-independent base-pairing of U2 snRNA to pre-mRNA (Figures 4 and 7; Supplementary Figure S7B).
- Under conditions permitting complex A to assemble, the numbers of all three components depend strongly on the number of 3΄ss (Figures 2, 3 and 5). The presence of ATP therefore confers stringency on binding, but all three components can bind prior to selection, as in route III (Figure 1).
- The levels of U2AF binding compared to U2 snRNP in complex A conditions depend on the pre-mRNA (Figures 2 and 5).
- Concurrent occupancy of candidate 3΄ss, as in routes II or III, leads to slower splicing (Supplementary Figure S6).
- With two introns and assembly stalled at complex A, the number of U2 snRNPs bound may or may not be restricted to a single snRNP, depending on the pre-mRNA, but binding by U1 snRNP (64), U2AF35 or U2AF65 is not restricted (Figures 3 and 7). This demonstrates that U2 snRNP recruitment can be regulated independently of U2AF.
- Both U2AF and U2 snRNPs show super-stoichiometric association in the absence of functional U1 snRNP and an intact 3΄ss (Figure 6). This suggests that the intrinsic affinity for the pre-mRNA is not limiting, as suggested by route I, but that stoichiometry is imposed by association of the splice sites and the formation of complexes by phosphorylated proteins.
- The results with examples of regulated splicing confirm that single molecule methods can recapitulate quantitative inferences from ensemble methods. Moreover, we confirm that U2AF and PTB compete to regulate *Tpm1* exon 3, and that U2 binding rather than U2AF binding limits the splicing of SMN2 exon 7 (Figure 7).

**Figure 8.**
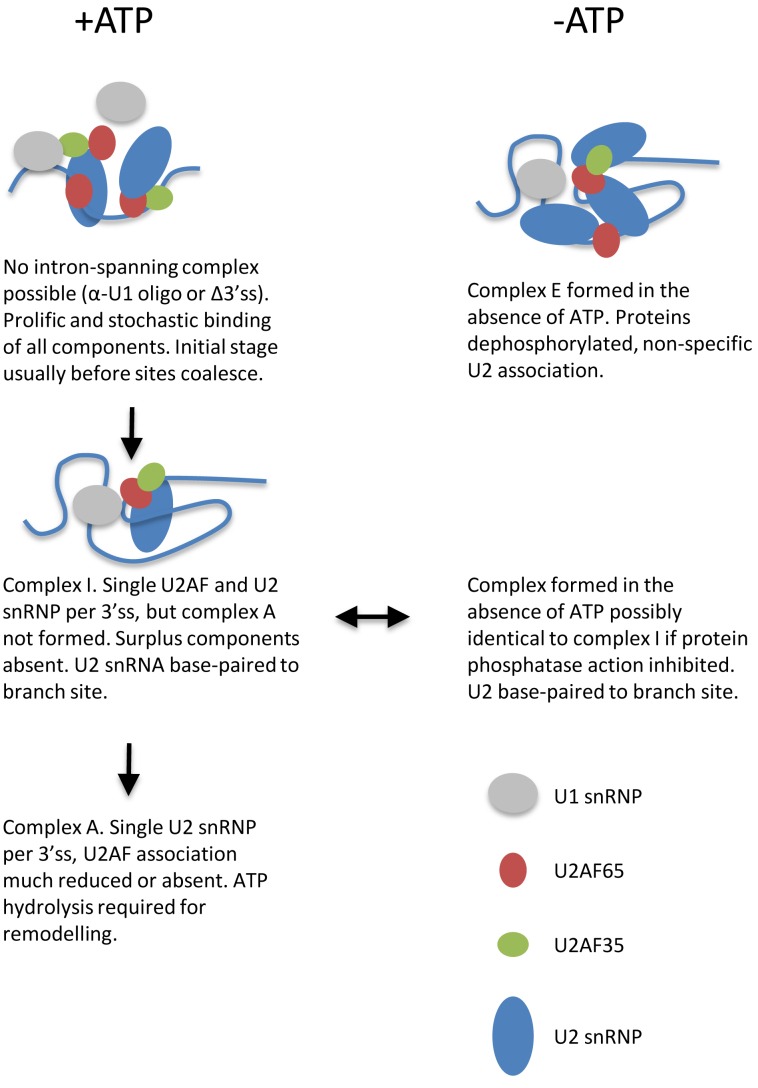
Scheme to show possible relationships between complexes. It is likely that sites are selected by affinity and kinetics: the first site to interact with the 5΄ss triggers complex I formation and molecules of U2AF or U2 snRNPs bound elsewhere are displaced. Binding is not limited by any intrinsic process to a single U2AF or U2 snRNP and so several strong sites could be occupied concurrently, as in routes II or III, and the complexes resist displacement. We note that there might be processes linked to transcription *in vivo* that reduce the chance of additional molecules of U2AF35 binding.

Three different patterns of association could be recognized in the presence of ATP under conditions preventing progression beyond complex A. After U1 inactivation or in the absence of 3΄ss sequences, conditions that prevent the formation of functional splicing complexes, there is super-stoichiometric but efficient association of U2AF35, U2AF65 and U2 snRNPs (Figure 6). With substrates that can splice, but only very poorly, and do not accumulate stable complex A, there is stoichiometric binding of the components relative to the 3΄ss but the level of association with U2 snRNPs is low (Figure 5). Finally, with substrates containing strong sites, the components bind stoichiometrically but the level of association with U2AF is lower than with U2 snRNPs (Figures 2, 3 and 7B). We suggest that these represent three stages of assembly (Figure 8): (i) stochastic and independent binding with no discrete complex formation or fixed stoichiometry relative to the 3΄ss, (ii) formation of pre-A complexes (which we term I, for initial) in which 5΄ss and 3΄ss participate and components bind stoichiometrically but U2AF binding levels are high and (iii) complex A, the hallmark of which is relatively low binding of U2AF. The level of U2AF is so low in some of the last cases that it is possible that it represents residual complex I and that *bona fide* complex A does not contain U2AF.

In the absence of ATP, under conditions in which complex E forms, occupancy by U2AF35 is limited to only one molecule per 3΄ss, as it is in the presence of ATP, but the association of U2AF65 is not so limited and U2 snRNP association appears to be relaxed. It is likely that some of the U2 snRNPs are associated with sequences other than the branchpoint. In previous studies, the proximity of the 5΄ end of U2 snRNA in complex E to the 5΄ss and 3΄ss and to the U1 snRNP was interpreted, on the basis that only one U2 snRNP was bound, to mean that these sites are clustered (22). However, our results suggest instead that U2 snRNPs might be associated with these sequences and that the splice sites are not in close proximity in complex E. This might be consistent with the observation that, while complex E is committed to splicing, the splice sites themselves have not been definitively selected at this stage (13,14,23).

There were striking changes if the depletion of ATP was done in the presence of phosphatase inhibitors. First, there was stoichiometric association of U2 snRNPs; the binding of excess U2 snRNPs had been prevented (Figures 4, 5 and 7). Taken with the relatively high level of colocalization with U2AF, these complexes closely resemble those described above as complex I. The effect of phosphatase inhibition might be linked to the phosphorylation of proteins known to be associated with U2 snRNP recruitment, such as SF1/mBBP (32,33), the mammalian branchpoint binding protein, or SF3B1 (82,83), a component of the U2 snRNP. It is possible that complex E does not reflect the early stages of complex assembly and that maintaining protein phosphorylation and stoichiometric binding provides a better model for complexes that might form normally prior to ATP hydrolysis.

A second striking feature of complexes formed after ATP depletion but in the presence of phosphatase inhibitors was the existence of AMT-psoralen cross-linking of U2 snRNA to the pre-mRNA (Supplementary Figure S7B). Such cross-links are characteristic of base-paired sequences (84–86). In complex E conditions, U2 snRNP is associated loosely and cross-linking has not been detected (17,19) (Supplementary Figure S8B), whereas U2 snRNA base-pairs and can be cross-linked in complex A. This has generally been taken to show that base-pairing requires ATPase activity. This is supported by experiments in yeast, in which Prp5 ATPase activity is required to disrupt a branchpoint-interacting stem-loop in U2 snRNA to enable full base-pairing to the branchpoint (87). However, the requirement for ATP can be by-passed in yeast by inactivation of Cus2 (88) and there is no evidence for a functional equivalent of Cus2 in mammals. The possibility that complex A might form without ATP hydrolysis has been raised previously (89). Our results suggest that the maintenance of protein phosphorylation enables U2 snRNA base-pairing, which matches the observation that there is no more than one U2 snRNP associated with each 3΄ss in such conditions. The ATPase activity associated with complex A formation might be involved in strengthening the interactions or displacement of U2AF.

Either the proportions of RNA molecules bound by the 3΄ss components or the level of occupancy of the RNA, or both, were increased by the ablation of U1 snRNP binding or mutations in binding sites. This is clearly at odds with both the expectation that binding would be limiting and the consequential recruitment models (Figure 1) that have dominated thinking about the association of splicing factors in the early stages of mammalian splicing. Previous work has relied heavily upon cross-linking to quantify the interactions of factors with RNA. The contrasting results may in large part arise from the difference between specific and tight binding interactions as opposed to those that are indirect and possibly mediated by other proteins. An interesting example is that of the U2 snRNP. It has not been clear whether the U1 snRNP is required to recruit U2 snRNP (39,69,70,90–96). However, it is required for tight interactions with the globin branchpoint sequence (Supplementary Figure S8). Our finding here that factors required for complex formation reduce U2 snRNP association, i.e. that complex formation imposes stringency and prevents super-stoichiometric association, is as far as we know unprecedented and could only have been revealed by single molecule methods.

To replace recruitment as a model for the mechanisms involved in forming the earliest splicing complexes, we favor the idea that the 3΄ss components bind prolifically but that formation of a defined complex takes place when all of the components are appropriately arranged and this triggers changes in the RNA that disrupt the association of surplus components. A critical role in this process is played by the U1 snRNP, even though this associates stringently and primarily at sites far from the 3΄ss. The ability of U1 snRNPs to stimulate displacement of the superfluous proteins is reminiscent of its ability to prevent polyadenylation over distances up to 1 kb from its binding sites (97–99). We have suggested previously that U1 snRNP binding triggers structural changes in the pre-mRNA that contribute to 5΄ss selection (64). We propose now that the interaction of correctly arranged 3΄ss components with the U1 snRNP (100) or SRSF1 (101) has a major role in triggering remodeling of pre-mRNA–protein complexes. This process must prevent the binding of surplus proteins that directly interact with the pre-mRNA, such as U2AF65, or those that probably associate indirectly, such as the U2 snRNP.

This model suggests that the less efficient splicing seen with multiple *bona fide* 3΄ss arises because tightly bound surplus U2AF35 or U2 snRNP complexes delay remodeling. It may be that there is no mechanism to prevent routes II and III but that they are nonetheless dead ends. In this regard, there is an interesting contrast with 5΄ss, where multiple occupancy of potential alternative sites by U1 snRNPs affects splice site selection but does not compromise splicing efficiency unless the sites are so close that there is steric inhibition (64,70). Splicing *in vivo* may benefit not from mechanisms to recruit factors but rather from mechanisms that delay multiple occupancy of candidate 3΄ss.

## Supplementary Material

Supplementary Data
