# Supplementary material for: Stoichiometries of U2AF35, U2AF65 and U2 snRNP reveal new early spliceosome assembly pathways
Source: Nucleic Acids Res. 2016 Sep 28;45(4):2051–67. doi: 10.1093/nar/gkw860 (PMC5389562; doi:10.1093/nar/gkw860)
Supplement: Supplementary Data [file gkw860_Supp.pdf]

Supplementary Data

Stoichiometries of U2AF35, U2AF65 and U2 snRNP reveal new early spliceosome assembly pathways

Li Chen[1], Robert Weinmeister[1], Jana Kralovicova[2], Lucy P. Eperon[1], Igor Vorechovsky[2], Andrew J. Hudson[3] and Ian C. Eperon[1,*]

[1]University of Leicester, Department of Molecular and Cell Biology, Leicester LE1 9HN, UK
[2]University of Southampton, Faculty of Medicine, Southampton SO16 6YD
[3]University of Leicester, Department of Chemistry, Leicester LE1 7RH, UK

Supplementary Figure 1

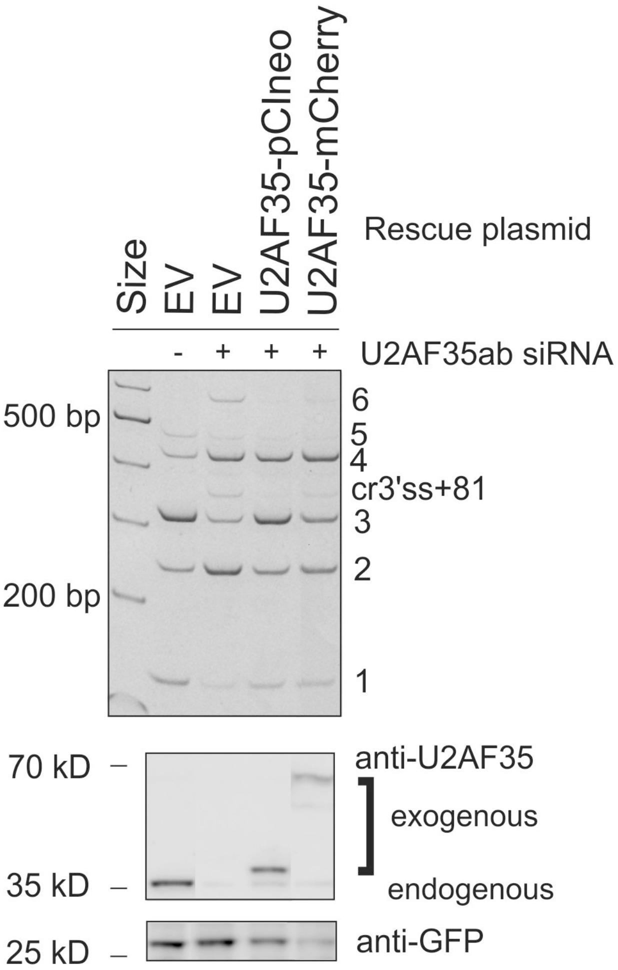

Supplementary Figure 2

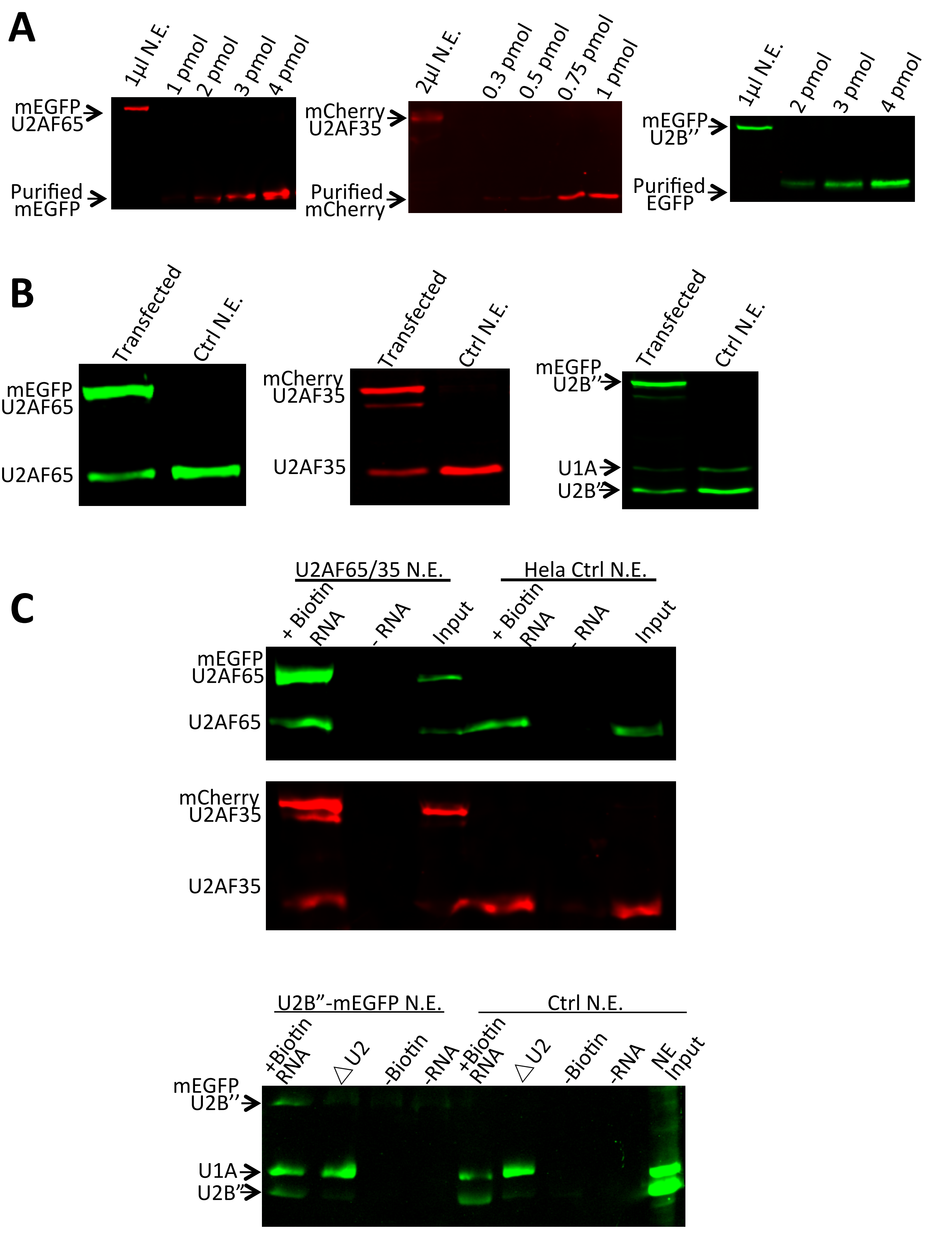

Supplementary Figure 3

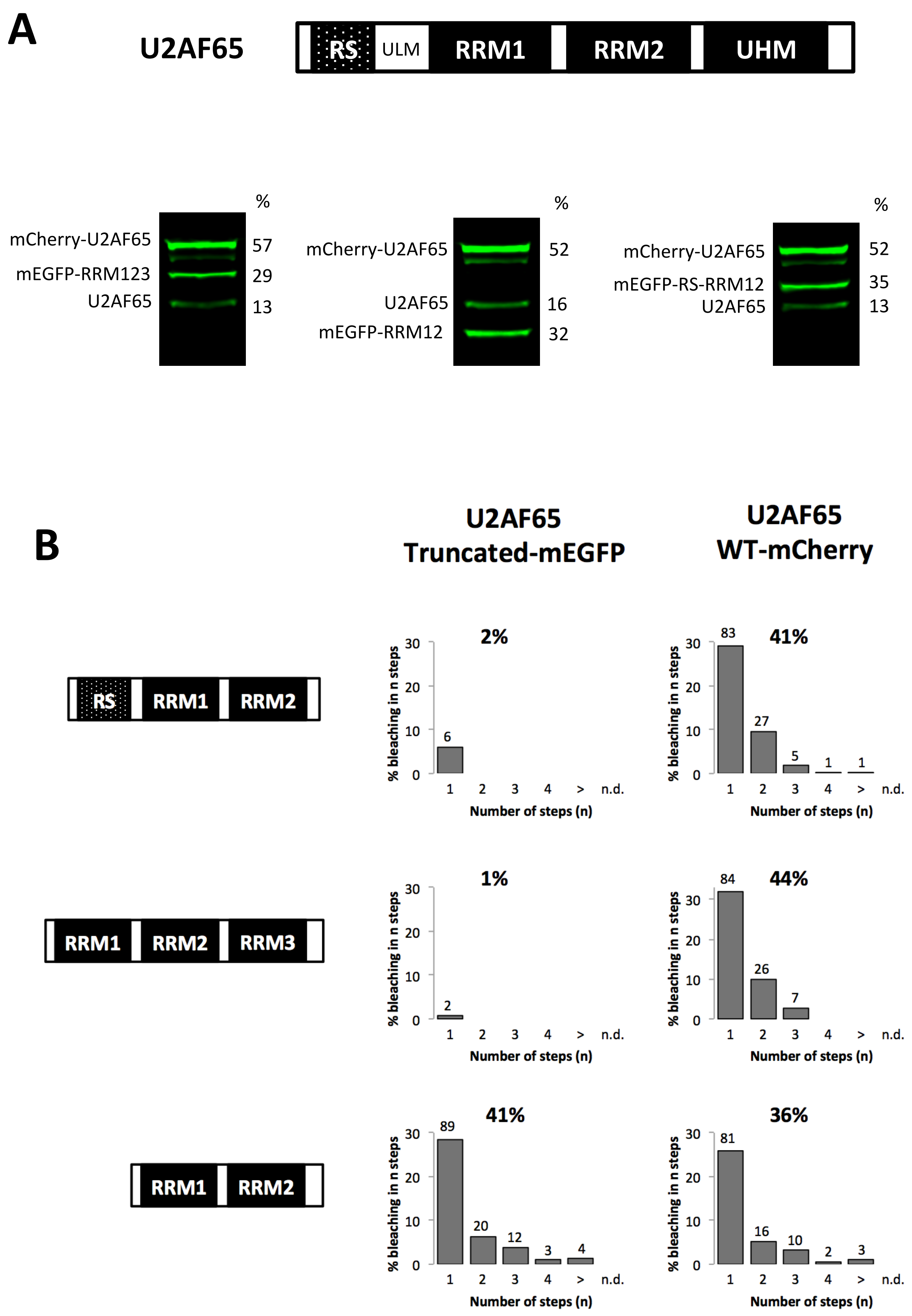

Supplementary Figure 4

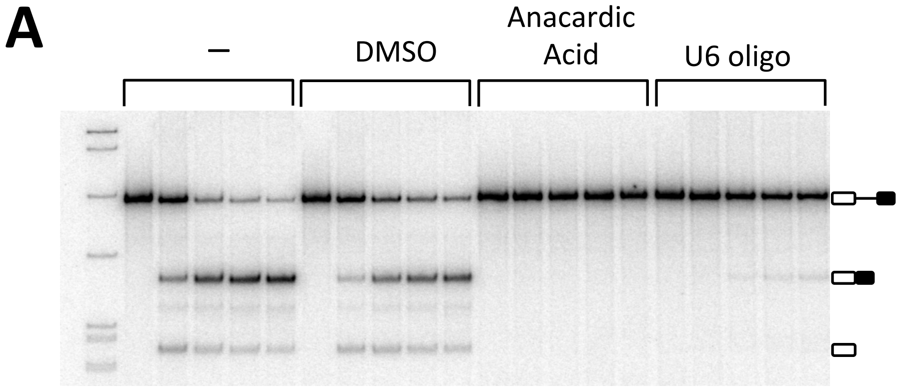

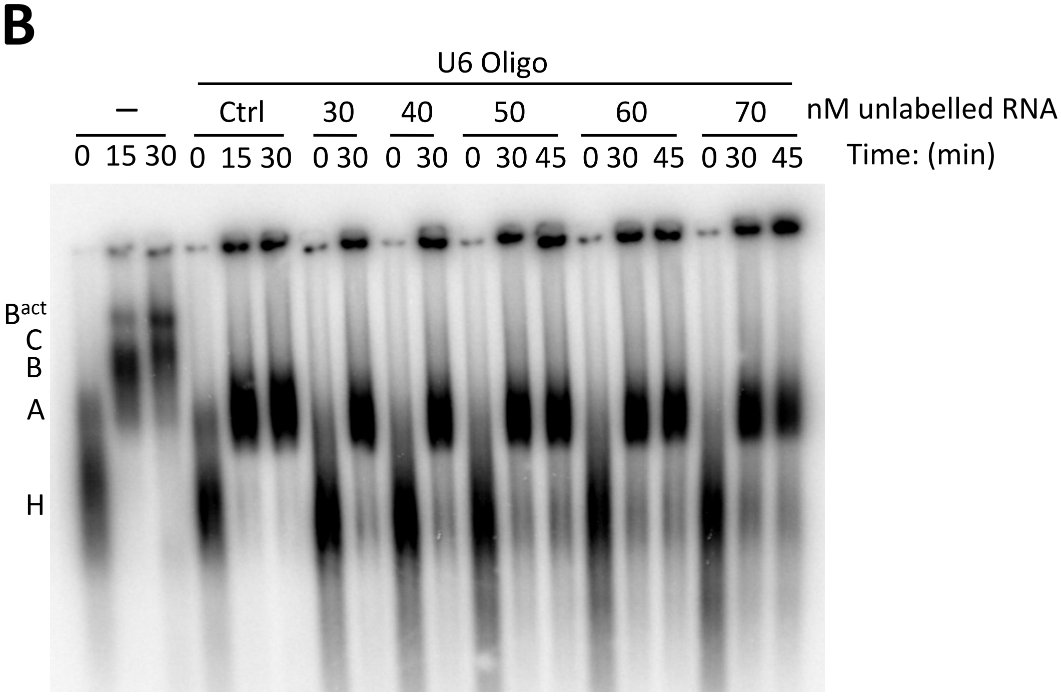

Supplementary Figure 5

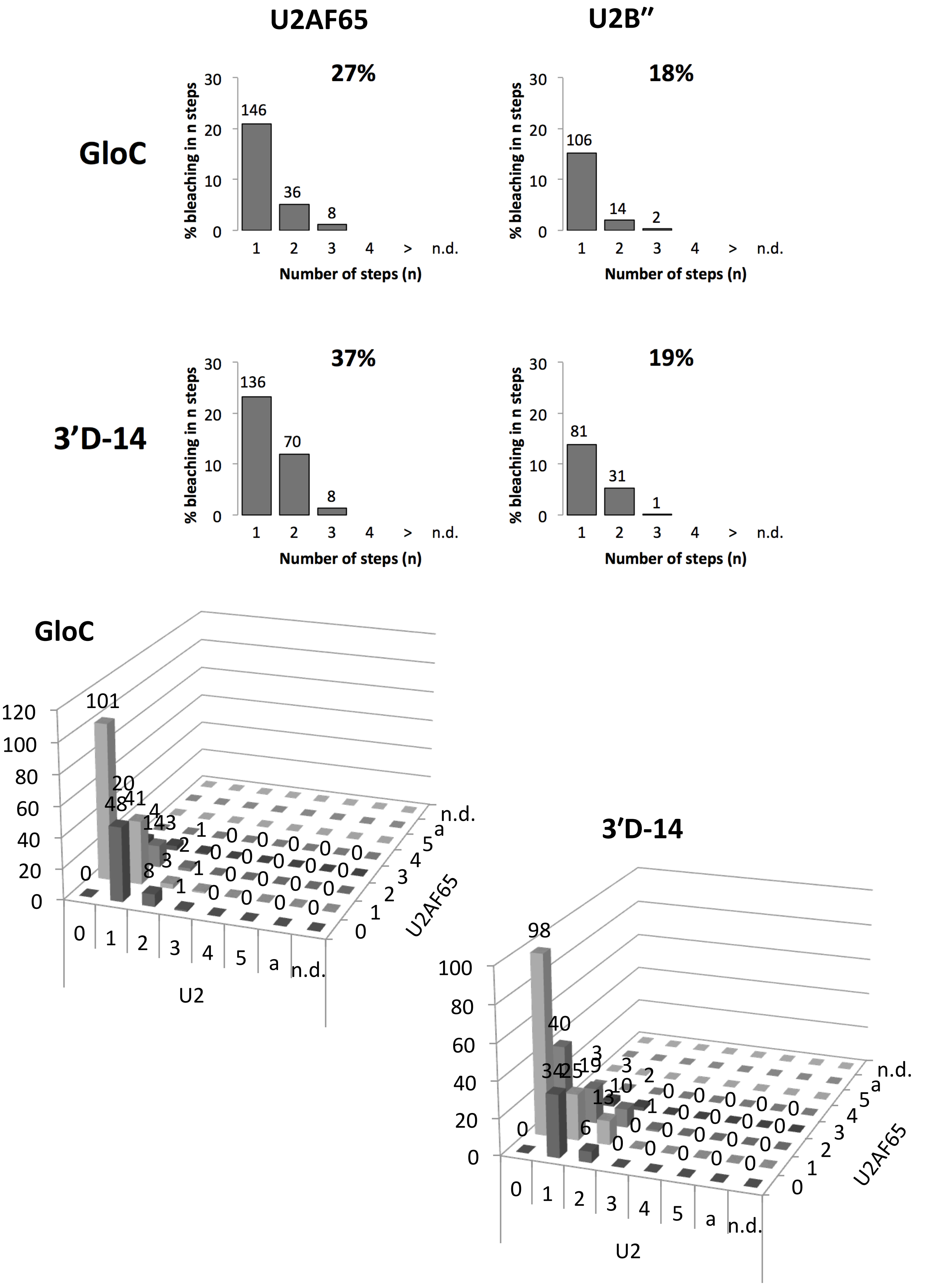

Supplementary Figure 6

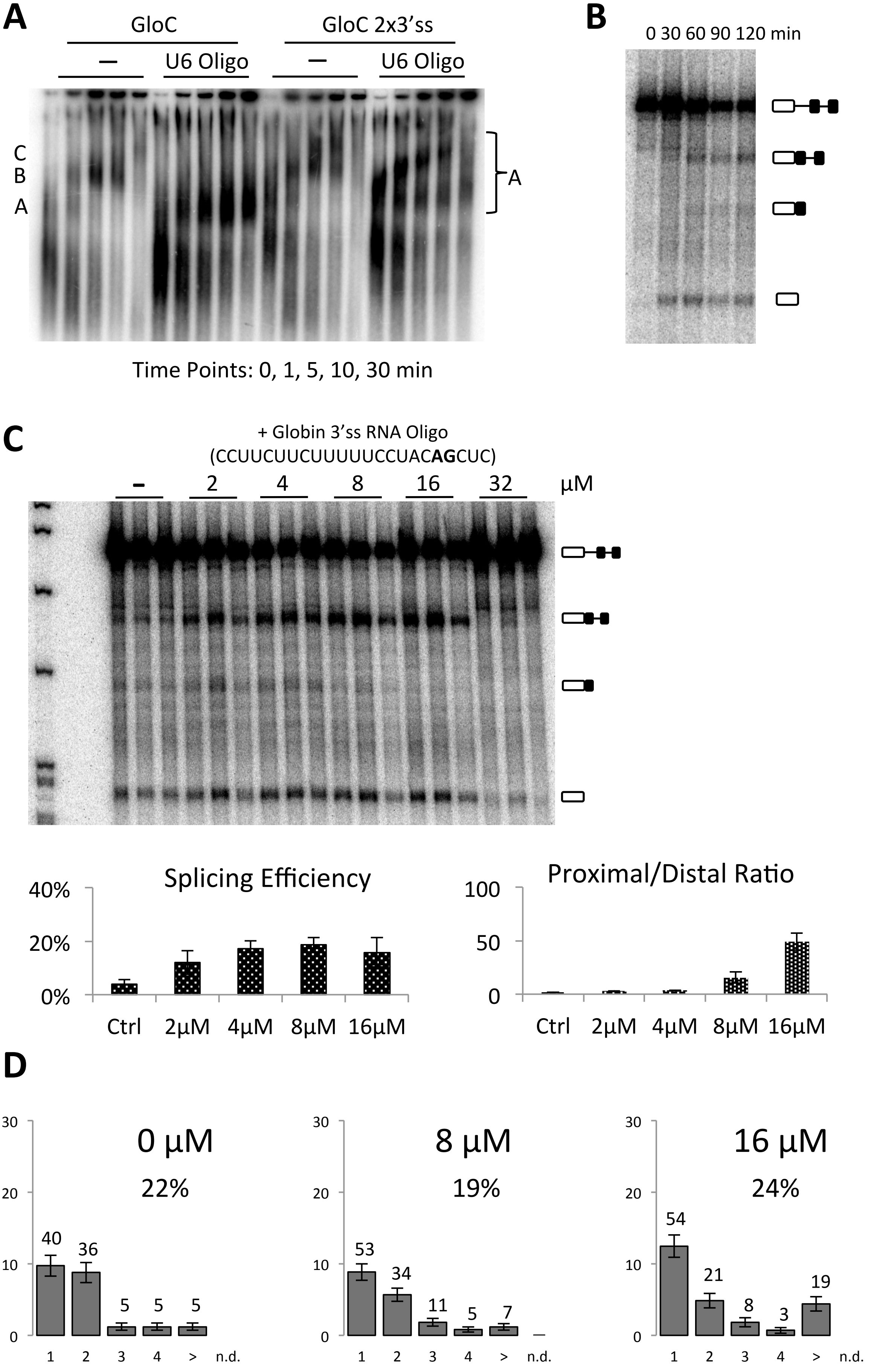

Supplementary Figure 7

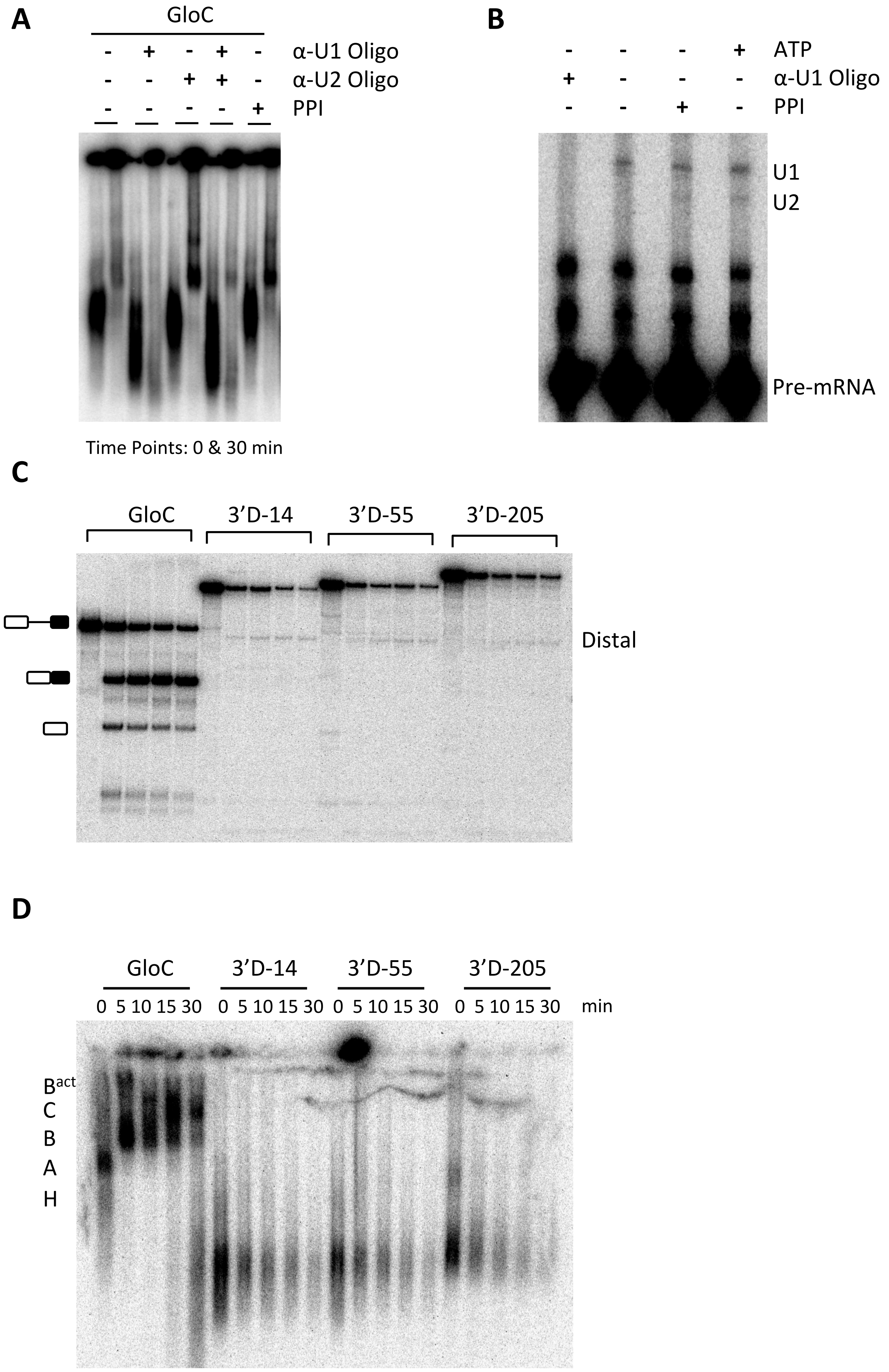

Supplementary Figure 8

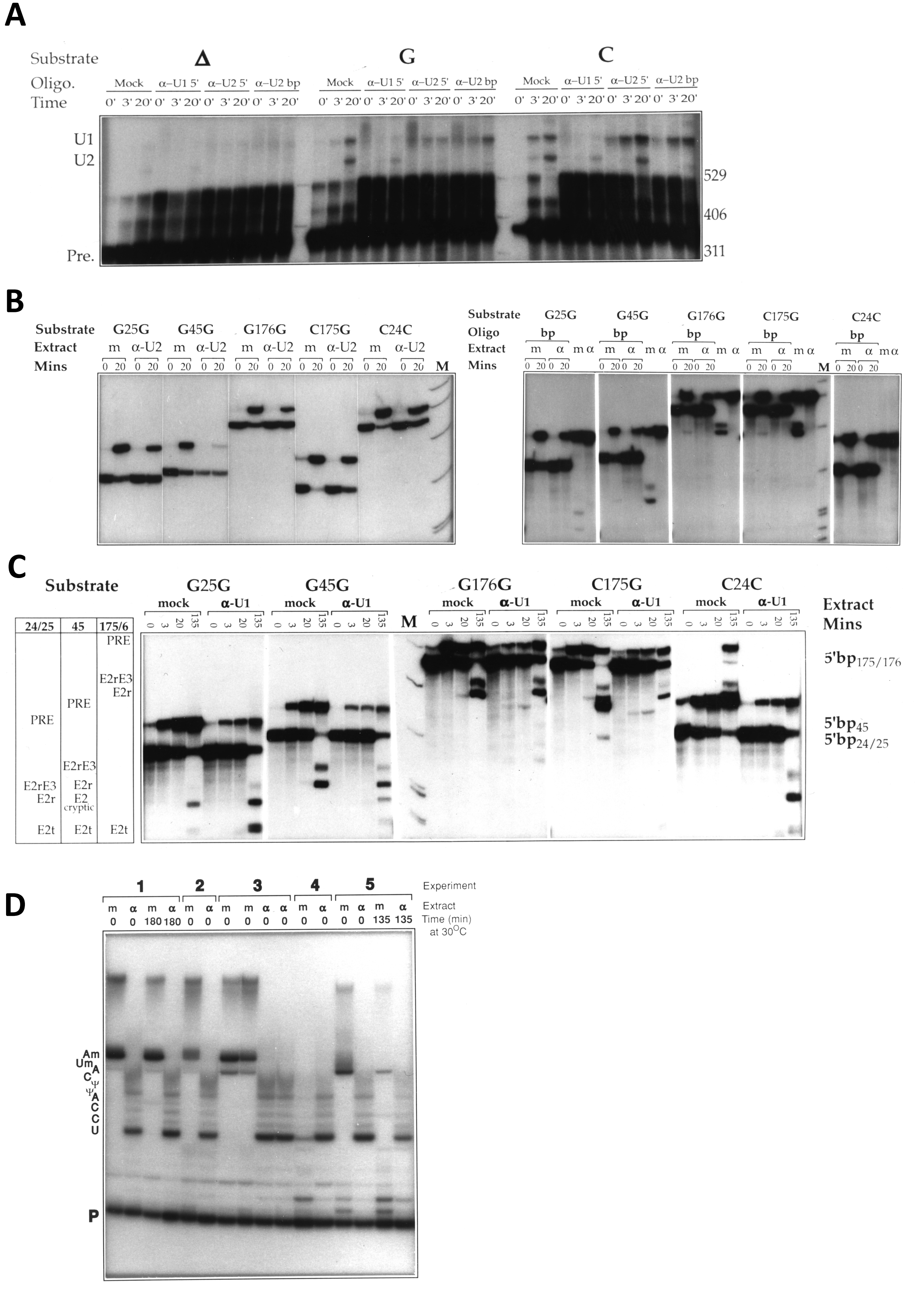

Supplementary Figure 9

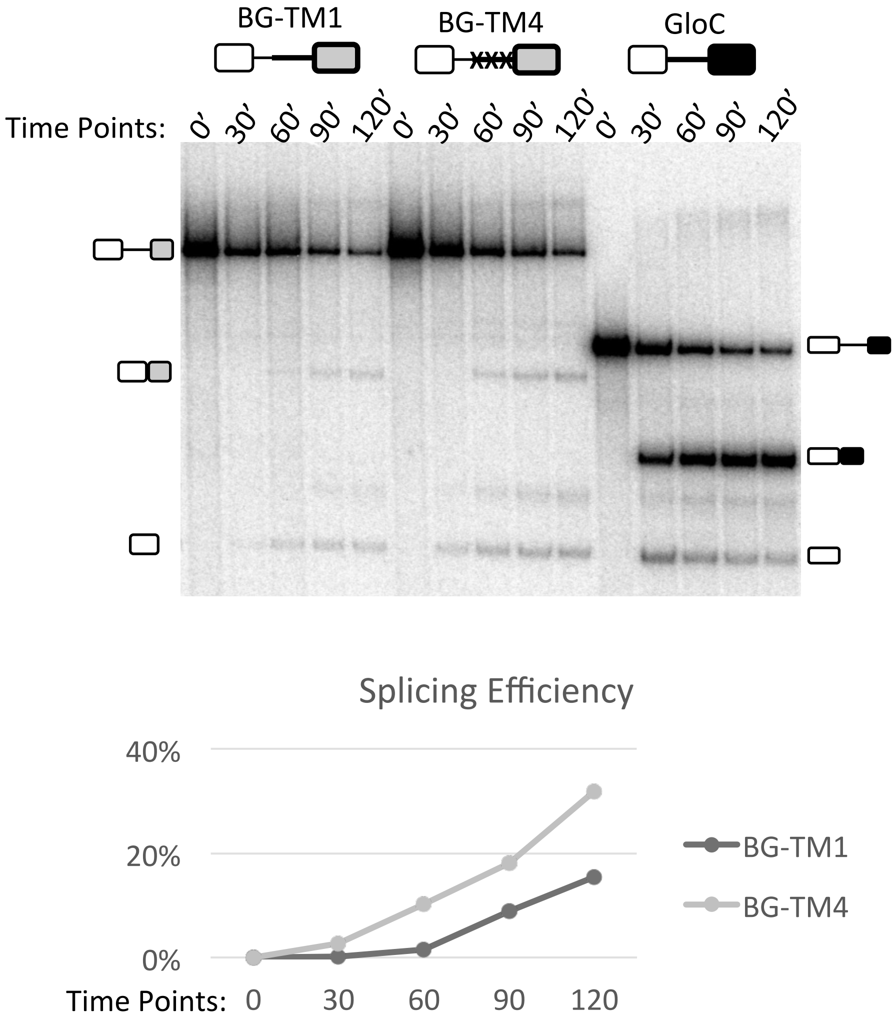

Legends for Supplementary Figures

Figure S1. Rescue of U2AF35-dependent splicing by exogeneous U2AF35. One hundred ng of reporter plasmids carrying the I-C haplotype of the human proinsulin gene (*INS*) (1) were cotransfected with 200 ng of rescue plasmids (pCI and mCherry) and 10 ng of pGFP into HEK293 cells (mock)-depleted of U2AF35. EV, empty vector; cr3′ss+81, a U2AF-dependent 3′ss activated 81 nucleotides downstream of authentic 3′ss of *INS* intron 1. RNA products (denoted 1-6) were as described (1). Isoform 6 contains *INS* intron 1. The lower panel shows immunoblots with the indicated antibodies. A lower expression of the mCherry plasmid as compared to pCIneo was associated with less efficient reconstitution of the *INS* splicing pattern, particularly isoform 3.

Figure S2. Expression and binding of proteins fused to mCherry or mEGFP. (A) Approximate quantification of concentration of fusion proteins in nuclear extract by comparison with pure mEGFP protein by western blotting and detection with fluorescent secondary antibodies after incubation with anti-mCherry or anti-mEGFP antibodies. (B) Quantification of concentrations of fusion proteins compared with endogenous proteins by western blotting and detection of anti-U2AF35, anti-U2AF65 or anti-U2B" with fluorescent antibodies. Control nuclear extracts were from untransfected HeLa cells. (C) Detection by western blotting of fusion protein and endogenous protein bound to biotinylated RNA after incubation of the RNA in nuclear extracts, as shown, and recovery on avidin beads. Control lanes show input nuclear extracts and mock purifications done in the absence of added RNA (-RNA) or with non-biotinylated RNA (-Biotin). ΔU2, deoxyoligonucleotides E15 and L15 were added to degrade U2 snRNA, showing that binding of U2B'' is mediated via the snRNP.

Figure S3. Activities of mEGFP-U2AF65 mutants lacking specific domains. (A) Analysis by western blotting of expression of mutant mEGFP-U2AF65 and full-length mCherry-U2AF65, co-expressed in HEK293T cells. Fluorescent secondary antibody was used to detect antibodies directed against U2AF65. (B) Distributions of bleaching steps of the co-expressed fluorescent proteins colocalised with GloC RNA.

Figure S4. (A) Splicing reaction time courses in the presence of anacardic acid or a 2'-O-methyl oligonucleotide complementary to U6 snRNA, both of which cause assembly to stall at complex A. Anacardic acid was dissolved in DMSO. Timepoints were (left to right) 0, 30, 60, 90 and 120 mins. (B) Native gel electrophoresis in the presence of heparin to test the efficacy of the 2'-O'methyl oligonucleotide in stalling assembly at complex A. The – and Ctrl reactions contained RNA at ~1 nM; otherwise, concentrations were as shown.

Figure S5. Colocalization of mCherry-U2AF65 and mEGFP-U2B'' on GloC and 3'-D14 RNA. The proteins were co-expressed and a nuclear extract prepared. Reactions were incubated in ATP and a 2'-O-methyl oligonucleotide complementary to U6 snRNA. The two horizontal axes show the numbers of steps in which mEGFP-U2B'' and mCherry-U2AF65 bleached and the vertical axis indicates the number of complexes with pre-mRNA in which these bleaching steps were seen.

Figure S6. Splicing of pre-mRNA with two alternative and identical 3′ss. (A) Native gel electrophoresis of substrates with one (GloC) or two 3′ss after incubation in splicing conditions for the times shown. U6 oligo indicates conditions producing stalling at complex A. Complexes are indicated by letters. Two forms of complex A formed with the GloC 2X3′ss pre-mRNA, one co-migrating with the complex formed on GloC RNA and the other migrating more slowly. (B) Time course of splicing of Gloc 2x3′ss. (C) Effects on splicing efficiency and ss selection of including an RNA oligonucleotide containing the sequence of the 3′ss region. Splicing reactions were for 120 min. The level of splicing and the ratio of use of the two sites are shown as histograms. (D) Effects of including the 3'ss oligonucleotide on the association of mCherry-U2AF35 with GloC-2x3'ss RNA in extracts depleted of ATP. The frequencies of complexes bleaching in **n** steps are shown, together with the percentage of RNA molecules colocalized with mCherry-U2AF35 and the concentration of the oligonucleotide in the incubation with nuclear extract.

Figure S7. Effects of protein phosphatase inhibition and weak alternative 3′ss on complex formation and U2 base-pairing to the pre-mRNA. (A) Native gel electrophoresis, in the absence of heparin (2), of GloC complexes formed after depletion of ATP. Oligonucleotides complementary to U1 and U2 snRNA were used to confirm that formation of complex E by 30 min was dependent and independent, respectively, of the snRNAs. Protein phosphatase inhibitors were included where shown (PPI). (B) Cross-linking by AMT-psoralen of snRNA to radiolabelled GloC pre-mRNA. Samples were incubated in splicing reaction mixtures either after depletion of ATP or in conditions stalling assembly in complex A. The ratio of U2 to U1 cross-links was the same in the reactions lacking ATP but including PI as in reactions in which complex A formed. This was confirmed in a repeat experiment. (C) Time courses of splicing of pre-mRNAs based on β-globin intron 1 with duplicated 3′ss separated by 14, 55 or 205 nts. Times were 0, 30, 60, 90 and 120 min. (D) Native gel electrophoresis of complexes formed by the pre-mRNA substrates in (C) after incubation in splicing conditions for the times shown.

Figure S8. Stable association of U2 snRNP with the branchpoint depends on U1 snRNA. (A) U2 base-pairing depends on the presence of a 5' ss and the 5' end of U1 snRNA. Analysis by denaturing gel electrophoresis of psoralen cross-linking to U1 and U2 snRNA by three pre-mRNAs based on β-globin intron 2: Δ, in which the 5′ss had been deleted, G, with the natural 5′ss sequence and C, in which the 5′ss was replaced with a consensus 5′ss (3). α-U1 5′, α-U2 5′ and α-

U2 bp refer to oligonucleotides triggering RNase H degradation of the corresponding U snRNA (3). Pre-mRNA cross-linked to U1 and U2 snRNA migrate as indicated on the side. (B) Stable branchpoint association is the result of U2 snRNA binding. Effects of U2 snRNA cleavage by oligonucleotides α-U2 5′ or α-U2 bp, as shown, on protection of the branchpoint against RNase H degradation. Splicing reactions were incubated for 0 min or 20 min and then challenged with the oligonucleotide complementary to the branchpoint. M, mock cleavage. The substrate pre-mRNAs contained two 5′ss, either G or C, separated by the distances shown (3). (C) Stable U2 binding depends on the 5' end of U1 snRNA. Effects of cleavage of the 5′ end of U1 snRNA on branchpoint protection and splicing when initiated at the times indicated during splicing reactions. Splicing reaction products are shown on the left (PRE, pre-mRNA; E2r, 5′ exon intermediate of step 1 at the downstream 5′ss; E2t, 5′ exon intermediate after step 1 at the upstream 5′ss; E2rE3, mRNA); RNase H cleavage products are shown on the right. U1 cleavage produces effects of branchpoint protection that are at least equal to those produced by U2 cleavage. Activation of splicing by U1 cleavage on pre-mRNA with close alternative 5′ss has been described previously (3). (D) Incubation of nuclear extract with the α-U1 5' oligonucleotide truncates the 5' end of U1 snRNA. Analysis by reverse transcription of the sites of cleavage of U1 snRNA by RNase H directed by the α-U15′ oligonucleotide. The results are shown from experiments with 5 different nuclear extracts and after incubation in splicing conditions for the times shown after addition of the oligonucleotide. The positions in U1 snRNA corresponding to the reverse transcriptase stops are shown; P, primer.

Figure S9. Splicing of pre-mRNA substrates containing exon 3 of TPM1. Substrate TM1 contains the 3′ end of intron 2 and exon 3 of TPM1; substrate TM4 contains the same sequences with three single point mutations in PTB binding sites in the polypyrimidine tract. Both substrates carried the same mutations in the branch site to attenuate splicing.

References

1. Kralovicova, J. and Vorechovsky, I. (2010) Allele-specific recognition of the 3' splice site of INS intron 1. *Hum. Genet.*, **128**, 383-400.
2. Das, R. and Reed, R. (1999) Resolution of the mammalian E complex and the ATP-dependent spliceosomal complexes on native agarose mini-gels. *RNA*, **5**, 1504-1508.
3. Eperon, I.C., Ireland, D.C., Smith, R.A., Mayeda, A. and Krainer, A.R. (1993) Pathways for selection of 5' splice sites by U1 snRNPs and SF2/ASF. *EMBO J.*, **12**, 3607-3617.
